# Estimated impact from the withdrawal of primary care financial incentives on selected indicators of quality of care in Scotland: controlled interrupted time series analysis

**DOI:** 10.1136/bmj-2022-072098

**Published:** 2023-03-22

**Authors:** Daniel R Morales, Mark Minchin, Evangelos Kontopantelis, Martin Roland, Matt Sutton, Bruce Guthrie

**Affiliations:** 1 Division of Population Health and Genomics, University of Dundee, UK; 2 National Institute for Health and Care Excellence, Centre for Guidelines, Manchester, UK; 3 Division of Informatics, Imaging and Data Sciences, University of Manchester, Manchester, UK; 4 Department of Public Health and Primary Care, University of Cambridge, Cambridge, UK; 5 Division of Population Health, Health Services Research and Primary Care, University of Manchester, Manchester, UK; 6 Advanced Care Research Centre, Usher Institute, University of Edinburgh, Edinburgh, UK

## Abstract

**Objective:**

To determine whether the withdrawal of the Quality and Outcomes Framework (QOF) scheme in primary care in Scotland in 2016 had an impact on selected recorded quality of care, compared with England where the scheme continued.

**Design:**

Controlled interrupted time series regression analysis.

**Setting:**

General practices in Scotland and England.

**Participants:**

979 practices with 5 599 171 registered patients in Scotland, and 7921 practices with 56 270 628 registered patients in England in 2013-14, decreasing to 864 practices in Scotland and 6873 in England in 2018-19, mainly due to practice mergers.

**Main outcome measures:**

Changes in quality of care at one year and three years after withdrawal of QOF financial incentives in Scotland at the end of the 2015-16 financial year for 16 indicators (two complex processes, nine intermediate outcomes, and five treatments) measured annually for financial years from 2013-14 to 2018-19.

**Results:**

A significant decrease in performance was observed for 12 of the 16 quality of care indicators in Scotland one year after QOF was abolished and for 10 of the 16 indicators three years after QOF was abolished, compared with England. At three years, the absolute percentage point difference between Scotland and England was largest for recording (by tick box) of mental health care planning (−40.2 percentage points, 95% confidence interval −45.5 to −35.0) and diabetic foot screening (−22.8, −33.9 to −11.7). Substantial reductions were, however, also observed for intermediate outcomes, including blood pressure control in patients with peripheral arterial disease (−18.5, −22.1 to −14.9), stroke or transient ischaemic attack (−16.6, −20.6 to −12.7), hypertension (−13.7, −19.4 to −7.9), diabetes (−12.7, −15.0 to −12.4), or coronary heart disease (−12.8, −14.9 to −10.8), and for glycated haemoglobin control in people with HbA_1c_ levels ≤75 mmol/mol (−5.0, −8.4 to −1.5). No significant differences were observed between Scotland and England for influenza immunisation and antiplatelet or anticoagulant treatment for coronary heart disease three years after withdrawal of incentives.

**Conclusion:**

The abolition of financial incentives in Scotland was associated with reductions in recorded quality of care for most performance indicators. Changes to pay for performance should be carefully designed and implemented to monitor and respond to any reductions in care quality.

## Introduction

Pay-for-performance schemes have been implemented in many healthcare systems in both high income and low-middle income countries.^1^ ^2^ ^3^ Some evidence suggests that pay for performance improves quality of care when introduced, although improvements are only consistently seen for process indicators, are variable between studies, and are typically small at best.^1^ ^3^ The Quality and Outcomes Framework (QOF) pay-for-performance system for primary care in the National Health Service was introduced in 2004 in all four UK countries (England, Scotland, Wales, and Northern Ireland).^4^ QOF provided financial incentives for a large number of quality of care indicators aimed at improving the clinical management of chronic diseases, with incentives paid to ensure that a certain proportion of patients on a number of chronic disease registers achieved performance targets. Over time, the scale and scope of financial incentives in QOF was reduced, with fewer indicators incentivised and smaller proportions of general practice income dependent on pay for performance, reflecting concerns about the costs of documenting quality of care without actually improving it, and other unintended adverse consequences, such as neglect of non-incentivised activity.^5^ ^6^ When QOF was introduced across the UK in 2004, there were 66 clinical quality indicators across 12 domains (eg, diabetes, cancer, and cervical smear tests), along with 56 organisational indicators (eg, relating to medicine management systems). Progressively, almost all organisational indicators have been removed, as where many clinical process indicators (eg, incentives to measure blood pressure) were perceived to be ineffective and unnecessary.^7^ Simultaneously new domains were added (eg, osteoporosis, depression), with 54 clinical quality indicators across 20 domains incentivised in England in financial year 2015-16. The choice and design of indicators were also increasingly diverging between the UK countries. These changes were consistent with the recommendations of many designers of pay-for performance programmes to refresh incentives to ensure that they were targeted at areas where performance was poor and that they should be withdrawn once improvement was sustained.^1^

Neither the implementation nor the withdrawal of QOF underwent a planned robust evaluation, however, and relatively few studies have investigated the effect of withdrawal of incentives on quality of care. Consistent with economic theory that targeting external motivation with incentives may crowd out internal professional motivation to improve care,^8^ qualitative research with providers in high income^9^ and low income^10^ ^11^ countries found that incentive withdrawal is perceived to have negative consequences on motivation and quality. The results of studies examining this association quantitatively have been mixed, with some studies finding no decline in quality^12^ ^13^ ^14^ ^15^ ^16^ ^17^ but others observing a worsening of quality after the removal of incentives,^18^ ^19^ ^20^ ^21^ ^22^ ^23^ ^24^ often back to similar quality levels before incentivisation or sometimes to lower levels.^18^ ^19^ ^20^ ^22^ ^24^

Three studies examined the effect of incentive withdrawal on quality of care in QOF. One of these studies examined changes in eight indicators that were incentivised from 2004 to 2006 and found no difference in quality of care after withdrawal of the incentives up to 2012.^16^ Seven of the eight indicators were, however, process indicators where the process remained partially incentivised in a matching intermediate outcome indicator (for example, incentives to monitor blood pressure in people with coronary heart disease were withdrawn, but practices were still incentivised to control blood pressure in a way that indirectly incentivised blood pressure measurement). In contrast, a study of England-wide incentives for screening of alcohol problem drinking observed no benefit from the introduction of incentivisation in 2008 but a rapid decline in screening to below 2008 levels when incentives were withdrawn in 2015.^24^ The remaining study examined the impact of withdrawing incentives for 12 indicators in England in 2014 and found that documented quality decreased for all indicators in the first year after financial incentives were removed, with reductions generally being largest for indicators related to documenting the provision of health advice.^23^

All three studies used time series methods to examine indicators that had been specifically targeted for removal, either because quality was believed to be high and stable or because the indicator was considered to be measuring care that was less important than other potential indicators. Such findings may not be generalisable to the overall question of what happens to performance when financial incentives are withdrawn. Although the NHS is a universal healthcare system across the UK regions, governance and decision making is devolved to country level. In April 2016, Scotland abolished the QOF but continued collecting national data on performance for a subset of QOF indicators. This abolition was done to reduce the bureaucratic burden on general practitioners and to free-up their time for patients. The abolition of QOF in Scotland created a natural experiment to compare indicators that were consistently measured in Scotland and England (where incentives were maintained) before and after April 2016. We evaluated the impact of QOF withdrawal on the quality of care in Scotland across a range of indicators, compared with changes in quality of care in England in the same period.

## Methods

### Data sources

For this controlled interrupted time series analysis of population level data for 16 quality of care indicators we extracted data on QOF primary care indicators in Scotland and England from the electronic medical records of family practices in both countries using UK-wide data specification. Data were extracted and reported annually for financial years April to March, and additionally in Scotland were collected for three financial years after the withdrawal of financial incentives (2016-17, 2017-18, and 2018-19). All data consist of practice level aggregates and are non-disclosive at patient level. English and Scottish QOF data are published and available for download from NHS Digital and Public Health Scotland, respectively. Scottish data post-QOF were collected as part of the transitional quality arrangements and were obtained from Public Health Scotland. Analysis uses population quality (proportion of people with the condition receiving the specified care or achieving the specified target) rather than payment quality where patients are excluded if unsuitable.

### Indicator selection and definition

We examined 16 of 25 potential quality of care indicators available during the transitional quality arrangements that were incentivised in both Scotland and England. These covered the three years before and after the withdrawal of QOF in Scotland until the end of the 2018-19 financial year. The 16 indicators included two that required affirmation by tick box to indicate that complex processes had been delivered (completion of a care plan in people with serious mental illness, and comprehensive foot screening in people with diabetes), nine intermediate outcome indicators (blood pressure control in people with peripheral arterial disease, stroke or transient ischaemic attack, hypertension, coronary heart disease, or diabetes), two indicators for different thresholds of blood pressure (≤150/90 mm Hg and ≤140/80 mm Hg), and three indicators of glycaemic control in people with diabetes (HbA_1c_ (glycated haemoglobin) thresholds ≤75 mmol/mol, ≤64 mmol/mol, and ≤59 mmol/mol), and five treatment indicators (influenza immunisation in people with stroke or transient ischaemic attack, chronic obstructive pulmonary disease (COPD), coronary heart disease, or diabetes, and antithrombotic (antiplatelet or anticoagulant) treatment in people with coronary heart disease). See table 1 for full details of indicators.

**Table 1:** List of included Quality of Outcomes and Framework performance indicators

Indicator identifier	Code (formerly)	Indicator type	Indicator description
**Complex processes**			
Mental health care planning	MH02 (MH002(S))	Complex process recording	Percentage of patients with schizophrenia, bipolar affective disorder, and other psychoses who have a comprehensive care plan documented in the record (in preceding 15 months), agreed between individuals, their family, or carers as appropriate
Diabetic foot screening	DM12 (DM012(S))	Complex process recording	Percentage of patients with diabetes, on the register, with a record of a foot examination and risk classification: low risk (normal sensation, palpable pulses), increased risk (neuropathy or absent pulses), high risk (neuropathy or absent pulses plus deformity or skin changes in previous ulcer), or ulcerated foot within preceding 15 months
**Intermediate outcomes**			
Blood pressure ≤150/90 mm Hg:			
Peripheral arterial disease	PAD02 (PAD002(S))	Intermediate outcome	Percentage of patients with peripheral arterial disease in whom the last blood pressure reading (measured in preceding 15 months) is ≤150/90 mm Hg
Stroke or transient ischaemic attack	STIA03 (STIA003(S))	Intermediate outcome	Percentage of patients with a history of stroke or transient ischaemic attack in whom the last blood pressure reading (measured in preceding 15 months) is ≤150/90 mm Hg
Hypertension	HYP06 (HYP006(S))	Intermediate outcome	Percentage of patients with hypertension in whom the last blood pressure reading (measured in preceding 12 months) is ≤150/90 mm Hg
Coronary heart disease	CHD02 (CHD002(S))	Intermediate outcome	Percentage of patients with coronary heart disease in whom the last blood pressure reading (measured in preceding 15 months) is ≤150/90 mm Hg
Diabetes	DM02 (DM002(S))	Intermediate outcome	Percentage of patients with diabetes, on the register, in whom the last blood pressure reading (measured in preceding 15 months) is ≤150/90 mm Hg
Blood pressure ≤140/80 mm Hg:			
Diabetes	DM03 (DM003(S))	Intermediate outcome	Percentage of patients with diabetes, on the register, in whom the last blood pressure reading (measured in preceding 15 months) is ≤140/80 mm Hg
HbA_1c_ (mmol/mol):			
≤75	DM09 (DM009(S))	Intermediate outcome	Percentage of patients with diabetes, on the register, in whom the last IFCC-HbA_1c_ is ≤75 mmol/mol in preceding 15 months
≤64	DM08 (DM008(S))	Intermediate outcome	Percentage of patients with diabetes, on the register, in whom the last IFCC-HbA_1c_ is ≤64 mmol/mol in preceding 15 months
≤59	DM07 (DM007(S))	Intermediate outcome	Percentage of patients with diabetes, on the register, in whom the last IFCC-HbA_1c_ is ≤59 mmol/mol in preceding 15 months
**Treatments**			
Influenza immunisation:			
Stroke or transient ischaemic attack	STIA09 (STIA009(S))	Treatment	Percentage of patients with stroke or transient ischaemic attack who have had influenza immunisation in preceding 1 August to 31 March
COPD	COPD07 (COPD007(S))	Treatment	Percentage of patients with COPD who have had influenza immunisation in preceding 1 August to 31 March
Coronary heart disease	CHD07 (CHD007(S))	Treatment	Percentage of patients with coronary heart disease who have had influenza immunisation in preceding 1 August to 31 March
Diabetes	DM18 (DM018(S))	Treatment	Percentage of patients with diabetes, on the register, who have had influenza immunisation in preceding 1 August to 31 March
Antiplatelet/oral anticoagulants in coronary heart disease	CHD05 (CHD005(S))	Treatment	Percentage of patients with coronary heart disease with a record in preceding 15 months that aspirin, an alternative antiplatelet treatment, or an anticoagulant is being taken

COPD=chronic obstructive pulmonary disease; HbA_1c_=glycated haemoglobin; IFCC=International Federation of Clinical Chemistry.

### Statistical analysis

Data for the 16 indicators consisted of three annual measurements before and three annual measurements after the year financial incentives were withdrawn in Scotland. We defined performance as the percentage of patients on each disease register who were not excluded by automatic criteria, such as recent practice registration, who received the specified care. We plotted the time series to check the validity of the data and to confirm assumptions of linearity.

The focus of our study was the estimated change in quality of care performance one year and three years after 2015-16 compared with that expected based on the pre-intervention trend. We specified this in a stepwise approach. Initially we used single group analysis of trends in Scotland before and after withdrawal of financial incentives at the end of the 2015-16 financial year, using interrupted time series linear regression to estimate immediate changes in quality in 2016-17, and change in trend. In the subsequent primary analysis, we performed interrupted time series linear regression analysis for multiple groups using the itsa command in Stata to examine changes in recorded quality in Scotland relative to changes in England used as a control.^25^ We used this analysis to calculate absolute differences in documented quality of care in Scotland compared with England at three years after the removal of financial incentives. Analyses were conducted in Stata version 14.

### Patient and public involvement

Although no patients or members of the public were involved in the conduct of the study owing to covid-19 restrictions, the idea for the study was inspired by speaking to patients and healthcare professionals while working in primary care before the pandemic.

## Results

The analysis included data from 979 general practices with 5 599 171 registered patients in Scotland and 7921 practices with 56 270 628 registered patients in England in 2013-14; a decline in practice numbers to 864 in Scotland and 6873 in England in 2018-19 was mainly because of practice mergers.

### Single country analyses in Scotland

In the single country analyses, trends in quality of care for seven of the 16 indicators were shown to be declining before the removal of financial incentives in Scotland for three intermediate outcomes (in people with diabetes: blood pressure ≤150/90 mm Hg, HbA_1c_ ≤64 mmol/mol, and HbA_1c_ ≤59 mmol/mol) and for four treatment indicators (influenza immunisation in people with stroke or transient ischaemic attack, COPD, coronary heart disease, or diabetes), although absolute changes from year to year were small (table 2). For the remainder of the indicators, no significant trend was observed during the baseline period.

**Table 2:** Single group interrupted time series regression analysis for each Quality and Outcomes Framework (QOF) performance indicator in Scotland

Indicator	Code*	Baseline performance 2013-14 (%)	Trend before QOF withdrawal (percentage point change per year) (95% CI)	Step change in year after QOF withdrawal (percentage point change)† (95% CI)	Change in trend after QOF withdrawal (additional percentage point change per year) (95% CI)	End performance 2018-19 (%)
**Complex processes**						
Mental health care planning	MH02	64.9	−0.4 (−0.8 to 0.01)	−30.4 (−35.2 to −25.5)	−4.4 (−7.6 to −1.4)	24.3
Diabetic foot screening	DM12	80.0	1.0 (−3.0 to 5.0)	−12.6 (−22.5 to −2.8)	−4.5 (−9.2 to 0.1)	64.4
**Intermediate outcomes**						
Blood pressure ≤150/90 mm Hg:						
Peripheral arterial disease	PAD02	85.3	0.1 (−0.7 to 1.0)	−11.8 (−16.4 to −7.3)	−2.8 (−5.6 to −0.01)	69.0
Stroke or transient ischaemic attack	STIA03	85.0	−0.1 (−1.1 to 1.0)	−9.0 (−12.7 to −5.2)	−2.6 (−4.7 to −0.4)	71.2
Coronary heart disease	CHD02	87.6	−0.1 (−0.6 to 0.4)	−7.3 (−9.6 to −5.0)	−2.2 (−3.6 to −0.8)	75.9
Diabetes	DM02	85.7	−0.5 (−0.7 to −0.6)	−5.3 (−8.0 to −2.6)	−1.8 (−3.4 to −0.1)	77.0
Hypertension	HYP06	79.0	0.1 (−0.7 to 0.9)	−11.0 (−14.7 to −7.2)	−2.0 (−4.25 to 0.3)	65.2
Blood pressure ≤140/80 mm Hg:						
Diabetes	DM03	67.8	−0.8 (−0.9 to 0.6)	−6.8 (−9.6 to −4.0)	−1.8 (−3.6 to 0.03)	54.0
HbA_1c_ (mmol/mol):						
≤75	DM09	78.2	0.04 (−0.7 to 0.8)	−1.9 (−3.6 to −0.1)	−0.6 (−1.4 to 0.2)	75.6
≤64	DM08	65.9	−0.6 (−1.2 to −0.1)	−0.6 (−1.9 to 0.7)	−0.1 (−0.6 to 0.5)	62.2
≤59	DM07	57.2	−1.1 (−1.9 to −0.2)	0.3 (−1.7 to 2.3)	0.3 (−0.6 to 1.2)	52.9
**Treatments**						
Influenza immunisation:						
Stroke or transient ischaemic attack	STIA09	78.7	−1.0 (−1.5 to −0.6)	−3.5 (−5.4 to −1.6)	0.6 (−0.5 to 1.8)	71.2
COPD	COPD07	81.4	−1.4 (−1.6 to −1.16)	−3.2 (−5.1 to −1.3)	0.6 (−0.6 to 1.8)	72.4
Coronary heart disease	CHD07	82.1	−1.2 (−2.0 to −0.46)	−2.7 (−5.1 to −0.3)	0.6 (−0.8 to 1.9)	74.6
Diabetes	DM18	78.2	−1.4 (−1.9 to −1.0)	−3.0 (−5.8 to −0.2)	0.6 (−1.1 to 2.4)	69.0
Antiplatelet or oral anticoagulation in coronary heart disease	CHD05	91.7	0.1 (−0.6 to 0.8)	−0.9 (−2.5 to 0.6)	−0.7 (−1.4 to 0.01)	90.1

CI=confidence interval; COPD=chronic obstructive pulmonary disease; HbA_1c_=glycated haemoglobin.

* See table 1 for definitions of indicators.

† Step change=change at one year.

One year after the removal of financial incentives, decreases in quality were documented for 13 of the 16 indicators in Scotland. Reductions occurred in recording of both complex processes (mental health care planning and diabetic foot screening), seven intermediate outcomes (blood pressure ≤150/90 mm Hg in people with peripheral arterial disease, stroke or transient ischaemic attack, hypertension, coronary heart disease, and diabetes; blood pressure control ≤140/80 mm Hg in people with diabetes; and HbA_1c_ ≤75 mmol/mol), and four treatment indicators (influenza immunisation in people with stroke or transient ischaemic attack, COPD, coronary heart disease, or diabetes). Reductions at one year ranged from −30.4 percentage points (95% confidence interval −35.2 to −25.5) for mental health care planning to −1.9 (−3.6 to −0.1) for HbA_1c_ ≤75 mmol/mol.

A change to a negative trend occurred in five of the 16 indicators over the three year period (mental health care planning, and blood pressure ≤150/90 mm Hg in people with peripheral arterial disease, stroke or transient ischaemic attack, coronary heart disease, or diabetes). Negative trends ranged from −4.4 percentage point change per year (95% confidence interval −7.6 to −1.4) for mental health care planning to −1.8 percentage points per year (−3.4 to −0.1) for blood pressure ≤150/90 mm Hg in people with diabetes. Supplementary figure S1 shows the results of the single group trend analysis for Scotland and supplementary figure S2 shows the results for England.

### Multiple group analysis

In the multiple group analysis when data from England were included as control, statistically significant reductions were still observed in 12 of the 16 indicators in Scotland one year after removal of QOF (table 3). Large reductions were still observed in recording of both complex processes: mental health care planning (−31.0 percentage points, 95% confidence interval −35.0 to −27.1) and diabetic foot screening (−13.8, −20.4 to −7.2). Statistically significant reductions were also observed in eight intermediate outcomes: blood pressure ≤150/90 mm Hg in people with peripheral arterial disease, stroke or transient ischaemic attack, hypertension, coronary heart disease, or diabetes; blood pressure ≤140/80 in people with diabetes; and HbA_1c_ ≤75 mmol/mol or ≤64 mmol/mol. Statistically significant reductions in intermediate outcomes at one year ranged from −12.5 percentage points (−15.6 to −9.4) for blood pressure ≤150/90 mm Hg in people with peripheral arterial disease to −2.4 percentage points (−4.8 to −0.05) for HbA_1c_ ≤64 mmol/mol. Statistically significant reductions at one year were, however, only observed for two treatment indicators—influenza immunisation in people with stroke or transient ischaemic attack (−3.9, −6.9 to −0.9) or with COPD (−3.8, −6.9 to −0.8).

**Table 3:** Multiple group interrupted time series regression analysis for each Quality and Outcomes Framework (QOF) performance indicator in Scotland compared with England

Indicator	Code*	% (95% CI)		
		Change at 1 year post-QOF Scotland *v* England	Difference in trend post-QOF Scotland *v* England	Absolute difference between Scotland and England at 3 years
**Complex processes**				
Mental health care planning	MH02	−31.0 (−35.0 to −27.1)	−4.6 (−6.7 to −2.5)	−40.2 (−45.5 to −35.0)
Diabetic foot screening	DM12	−13.8 (−20.4 to −7.2)	−3.2 (−5.0 to −1.3)	−22.8 (−33.9 to −11.7)
**Intermediate outcomes**				
Blood pressure ≤150/90 mm Hg:				
Peripheral arterial disease	PAD02	−12.5 (−15.6 to −9.4)	−2.7 (−4.5 to −0.8)	−18.5 (−22.1 to −14.9)
Stroke or transient ischaemic attack	STIA03	−10.2 (13.0 to −7.4)	−2.4 (−3.8 to −1.1)	−16.6 (−20.6 to −12.7)
Hypertension	HYP06	−10.5 (−14.3 to −6.8)	−1.7 (−3.3 to −0.1)	−13.7 (−19.4 to −7.9)
Coronary heart disease	CHD02	−8.0 (−9.7 to −6.3)	−2.2 (−3.1 to −1.2)	−12.8 (−14.9 to −10.8)
Diabetes	DM02	−6.2 (−8.2 to −4.1)	−1.7 (−2.8 to −0.5)	−10.4 (−13.0 to −7.8)
Blood pressure ≤140/80 mm Hg:				
Diabetes	DM03	−7.8 (−10.1 to −5.6)	−2.4 (−3.9 to −1.0)	−12.7 (−15.0 to −10.4)
HbA_1c_ (mmol/mol):				
≤75	DM09	−3.2 (−5.4 to −0.9)	−0.4 (−1.4 to 0.5)	−5.0 (−8.4 to −1.5)
≤64	DM08	−2.4 (−4.8 to −0.05)	−0.5 (−1.6 to 0.7)	−3.4 (−6.7 to −0.03)
≤59	DM07	−1.9 (−4.5 to 0.8)	−0.5 (−1.8 to 0.9)	−2.1 (−5.7 to 1.6)
**Treatments**				
Influenza immunisation:				
Stroke or transient ischaemic attack	STIA09	−3.9 (−6.9 to −0.9)	−0.1 (−1.7 to 1.5)	−3.9 (−7.8 to 0.1)
COPD	COPD07	−3.8 (−6.9 to −0.8)	−0.2 (−1.9 to 1.5)	−3.4 (−7.3 to 0.4)
Coronary heart disease	CHD07	−3.2 (−6.3 to 0.03)	−0.2 (−1.8 to 1.4)	−3.2 (−7.6 to 1.2)
Diabetes	DM18	−3.3 (−6.9 to 0.2)	−0.01 (−1.8 to 1.8)	−2.4 (−7.2 to 2.5)
Antiplatelet or oral anticoagulation in coronary heart disease	CHD05	−0.8 (−1.8 to 0.3)	−0.4 (−0.4 to −0.3)	−1.4 (−3.3 to 0.6)

CI=confidence interval; COPD=chronic obstructive pulmonary disease; HbA_1c_=glycated haemoglobin.

**Fig 1:**
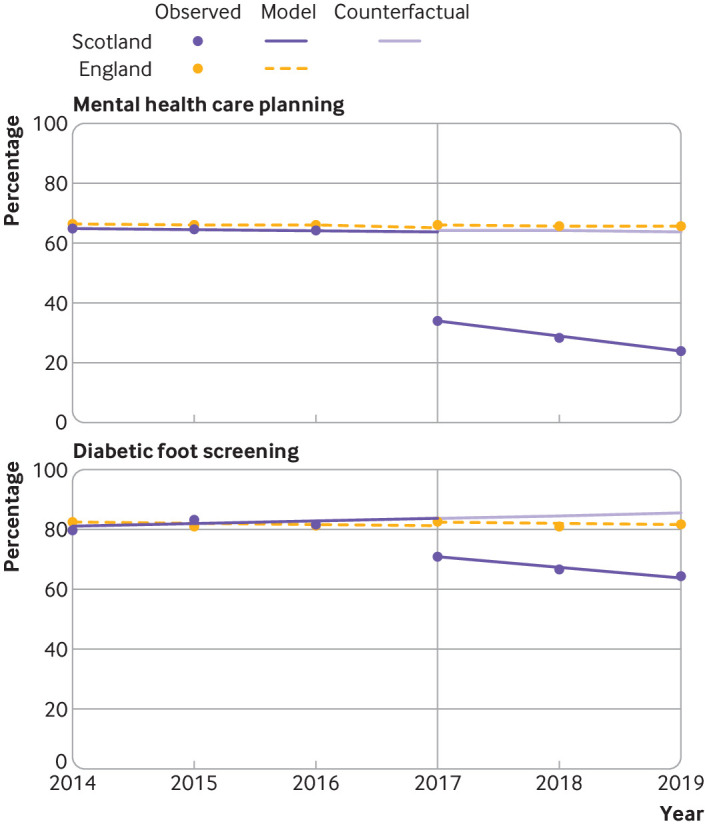
Multiple group interrupted time series analysis comparing indicators related to complex processes between Scotland and England. Vertical reference line indicates the first observation after withdrawal of the Quality and Outcomes Framework (QOF) at the end of the 2015-16 financial year in Scotland. The counterfactual for Scotland is based on Scottish prior trends and English immediate and post-QOF withdrawal effects. Regression was performed with Newey-West standard errors, and lag(0)

**Fig 2:**
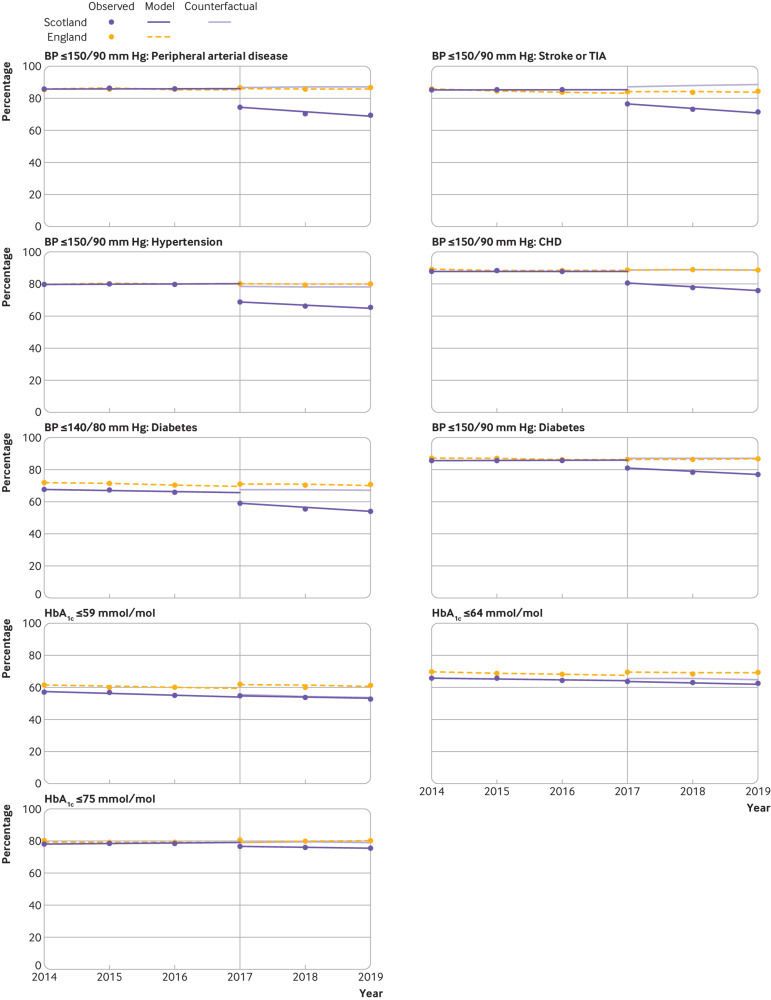
Multiple group interrupted time series analysis comparing indicators related to intermediate outcomes between Scotland and England. Vertical reference line indicates the first observation after withdrawal of the Quality and Outcomes Framework (QOF) at the end of the 2015-16 financial year in Scotland. The counterfactual for Scotland is based on Scottish prior trends and English immediate and post-QOF withdrawal effects. Regression was performed with Newey-West standard errors, and lag(0). BP=blood pressure; CHD=coronary heart disease; HbA_1c_=glycated haemoglobin; TIA=transient ischaemic attack

**Fig 3:**
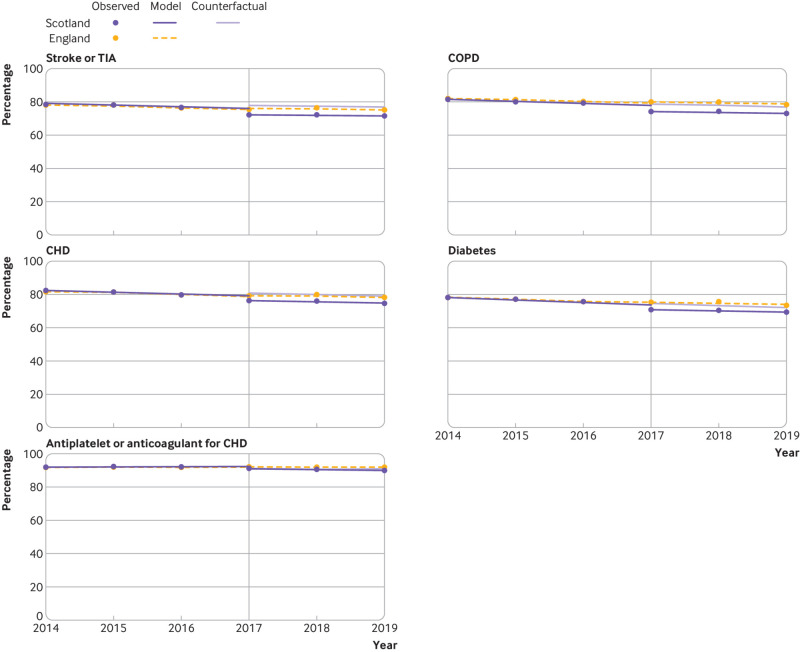
Multiple group interrupted time series analysis comparing treatment related indicators between Scotland and England. Vertical reference line indicates the first observation after withdrawal of the Quality and Outcomes Framework (QOF) at the end of the 2015-16 financial year in Scotland. The counterfactual for Scotland is based on Scottish prior trends and English immediate and post-QOF withdrawal effects. Regression was performed with Newey-West standard errors, and lag(0). CHD=coronary heart disease; COPD=chronic obstructive pulmonary disease; TIA=transient ischaemic attack

Compared with baseline trends, a statistically significant change to a negative trend was observed in nine indicators over the three year period in Scotland (two complex processes, six intermediate outcomes, and one treatment outcome) (table 3, fig 1, fig 2, and fig 3). Reductions ranged from −4.6 percentage point change per year (95% confidence interval −6.7 to −2.5) for mental health care planning to −0.4 percentage point change per year (−0.4 to −0.3) for antiplatelet or oral anticoagulant treatment in people with coronary heart disease.

The absolute percentage point difference between Scotland and England at three years after withdrawal of financial incentives was observed to be statistically significant for 10 indicators (two complex processes and eight intermediate outcomes). The largest reductions were observed for the two complex processes of mental health care planning (absolute difference in percentage points −40.2, 95% confidence interval −45.5 to −35.0) and diabetic foot screening (−22.8, −33.9 to −11.7). Substantial reductions were also observed in blood pressure related intermediate outcomes compared with England for ≤150/90 mm Hg in people with peripheral arterial disease (−18.5, −22.1 to −14.9), stroke or transient ischaemic attack (−16.6, −20.6 to −12.7), hypertension (−13.7, −19.4 to −7.9), or coronary heart disease (−12.8, −14.9 to −10.8), and blood pressure ≤140/80 mm Hg in people with diabetes (−12.7, −15.0 to −10.4). Reductions in HbA_1c_ intermediate outcomes were smaller but still observed to be significant for HbA_1c_ ≤75 mmol/mol (−5.0, −8.4 to −1.5) and ≤64 mmol/L (−3.4, −6.7 to −0.03) but not for HbA_1c_ ≤59 mmol/mol (−2.1, −5.7 to 1.6). At three years, no significant difference was observed for any of the treatment indicators between Scotland and England (table 3).

## Discussion

In time series analysis of Scotland-wide data, withdrawal of QOF financial incentives in 2016 was associated with a reduction in documented quality of care in 12 of the 16 indicators measured at one year, and with a negative change in trend over the three year period in nine of the 16 indicators compared with England (where incentives were maintained). Similarly, statistically significant reductions were also documented for quality of care in Scotland three years after withdrawal for 10 of the 16 indicators examined, which were large (>20 percentage point differences) for the two complex processes, large for blood pressure control (10-20 percentage point differences), and small for two of the three glycaemic control indicators (<5 percentage point differences). We found no statistically significant differences between Scotland and England for indicators of glycaemic control in diabetes, or for the treatment indicators at three years; although small, short term reductions were observed at one year.

### Strengths and limitations of this study

Two key strengths of the study are the use of interrupted time series analysis, which is a robust method for examining the impact of an intervention when randomisation is not possible,^26^ and the availability of population data from two countries where the indicators examined were incentivised in both countries before April 2016, but with incentives subsequently withdrawn in Scotland and maintained in England. Limitations are that the time series included relatively few time points (three years before and three years after the withdrawal of QOF) and precludes examination for the presence of autocorrelation. Follow-up duration is, however, constrained by the post-QOF Scottish data being collected for only three years and by the onset of the covid-19 pandemic. The size of each dataset also meant that there was relatively little noise (random variation) in the time series data, and the availability of an English control population increases confidence that the observed changes in Scotland were the result of withdrawal of financial incentives. A further limitation is that we restricted analysis to indicators that were implemented in both England and Scotland in the three years April 2013 to March 2016 and therefore our study does not cover the full range of indicators implemented in each country in this period. Reasons for this are because different indicator definitions were used or because QOF indicators might have been removed over time, such as the percentage of patients with myocardial infarction treated with an angiotensin converting enzyme inhibitor (or angiotensin II receptor blocker if intolerant to an angiotensin converting enzyme inhibitor), aspirin or an alternative antiplatelet treatment, β blocker, or statin, retired in April 2015 in England. The 16 indicators we examined represent 30% of the clinical indicators implemented in Scotland in financial year 2015-16, but they are weighted towards cardiovascular domains and might not be representative of all indicators. The findings are, however, consistent with those observed in published analysis of a different range of withdrawn indicators in England, and we believe the results are likely to be generalisable.^23^ The interrupted time series method assumes no other exogenous factors—in this case, whether another local or national policy change or intervention occurred during the study period. No other national interventions targeted the examined indicators, although we cannot rule out changes at local level (eg, clinical commissioning group or practice). Even if such changes had occurred, it is unlikely to explain the effect sizes we observed. Importantly, we examined changes in incentivised quality of care, which is only a subset of care processes and outcomes, and we did not examine the impact on non-incentivised or hard to measure care because the data are not available.

### Comparison with other studies

Several studies have documented declines in quality of care after the withdrawal of financial incentives,^18^ ^19^ ^20^ ^21^ ^22^ ^23^ ^24^ often back to levels similar to or worse than before the incentivisation.^18^ ^19^ ^20^ ^22^ ^24^ However, other studies have observed no decline in quality of care,^13^ ^14^ ^15^ ^16^ ^17^ although for one of these studies, care remained partially incentivised through other indicators for seven of the eight measures examined.^16^ Another study that followed-up a trial of incentives for diabetes care did not observe declines in quality when incentives were withdrawn, but the study also did not observe consistent improvements when incentives were introduced, making lack of change after withdrawal hard to interpret.^15^ ^27^ For the remaining three studies where quality did not decline when incentives were withdrawn, incentives were part of more comprehensive quality improvement interventions^13^ ^14^ ^17^ and it is plausible that other intervention components led to sustainability. Consistent with this finding, a factorial trial of short term (12 week) financial incentives, and training and support interventions for alcohol screening and intervention found sustained (nine month) benefit of training and support but not of incentives.^22^ Our study examined a larger set of indicators, and, as with previous analysis of England only data,^23^ we observed no decline in quality for some indicators and decline in quality for others.

Despite the observed changes in quality of care, it remains likely that some of the observed decline in Scotland relates to a reduction in documentation within the medical record, as opposed to care not being delivered. This is consistent with our observation that changes in treatment or glycaemic control were small and often not statistically significant, whereas changes in blood pressure control and the recording of complex process delivery by tick box were larger. A key difference between these types of indicators is that prescriptions and laboratory tests were reliably captured within the electronic medical record if they were done, because almost all prescriptions are created electronically, and practice requested laboratory tests are automatically entered into the record. In contrast, blood pressure measurements and complex processes can be recorded in free text as well in the coded fields counted in QOF indicators, and both are therefore more prone to gaming. For example, practices can decide which blood pressures to record as values (counted in indicators) versus in free text (visible for clinical care but not counted in indicators), and evidence suggests differences in the how blood pressures were documented when the thresholds for incentivised indicators were revised in the past.^12^ Changes in the recording of complex processes are even harder to interpret,^23^ since payment depends on ticking a box affirming that, for example, a care plan has been completed, but with no evidence required as to quality or completeness. The extent to which the large changes in complex process recording represent equally large reductions in quality of care is therefore uncertain. It is possible, however, that these differences might not be solely due to documentation because they are relatively time consuming, and it is possible that they may not be prioritised.

This difficulty in observing the actual effects of incentives is a dilemma for all evaluations of pay-for-performance schemes, where improvements in performance on introduction of the incentives and declines on their withdrawal may both be primarily driven by changes in documentation. We focused on treatment and glycaemic control indicators where documentation of performance is least likely to have an impact, and we concluded that withdrawal of financial incentives likely had small negative effects on actual quality of care.

### Policy implications

Our results suggest that removal of QOF in Scotland was associated with a reduction in documented quality of care for some but not all indicators, with variation in the relative size of changes and only small reductions in indicators that were least subject to changes in how practices document (as opposed to deliver) care. These findings are highly relevant to designers of pay-for-performance and healthcare quality improvement programmes internationally, as well as to post-covid-19 discussions about the future of QOF in the other UK countries.^28^ ^29^ Assuming that high levels of quality of incentivised care will be sustained after incentives are withdrawn is problematic, and so retaining the ability to evaluate what happens in pay-for-performance systems after their removal is critical. A key recommendation therefore should be that data continue to be collected for a period after the withdrawal of any indicator or performance scheme to monitor the impact, and ideally that data are collected in ways that minimise the effect of documentation. For example, evaluation of changes in recorded blood pressure would be usefully complemented by collection of data on the intensity of antihypertensive treatment. Responses to any observed changes would then be better based on evidence, allowing the targeting of quality improvement interventions (of which incentives are only one) where required.

Research examining what happens when incentives are withdrawn has largely focused on changes in incentivised measures, and more work is required to understand the actual impact on quality of incentivised care (for example, by teasing out changes in documentation and gaming from actual changes in care, for example) such as when examining prescriptions of antihypertensives as well as values of recorded blood pressure together with admissions for incident myocardial infarction in people with hypertension. Additionally, the introduction of incentives was associated with a negative impact on the quality of care for non-incentivised conditions,^5^ and evaluating quality of care more broadly would be invaluable, not least because the withdrawal of QOF incentives in Scotland was accompanied by the introduction of new approaches to quality improvement,^30^ and this new approach may have had positive effects on general practitioner satisfaction, recruitment, and retention.^31^ Although improvements in quality of care from the introduction of QOF appeared relatively small, evidence suggested that QOF reduced variation between practices and in particular narrowed the quality gap between practices serving socioeconomically deprived versus affluent practices.^5^ Research is needed to examine the impact on variation between practices and inequities of withdrawing incentives. Finally, randomised controlled trials are also needed into the effects of large scale incentive schemes with embedded process evaluation.

### Conclusion

Withdrawal of QOF in Scotland in 2016 compared with England where financial incentives were maintained was associated with reductions in recorded quality of care for 12 of 16 indicators after one year and 10 of 16 indicators after three years. Further research is needed to better understand the full impact of QOF withdrawal and the accompanying refocusing of quality improvement resources.

**What is already known on this topic**

When implemented in 2004 the UK Quality and Outcomes Framework (QOF) for primary care was the world’s largest healthcare pay-for-performance scheme
In 2016, Scotland abolished QOF financial incentives but continued to collect QOF quality indicator data for the next three years
Neither the implementation nor the withdrawal of QOF underwent a planned robust evaluation

**What this study adds**

In a controlled interrupted time series analysis, compared with England, the withdrawal of QOF in Scotland was associated with reductions in recorded quality of care for 12 of 16 indicators after one year and 10 of 16 indicators after three years
The largest changes at three years were observed for the recording, by tick box, of complex processes (mental health care planning and diabetic foot screening), but substantial reductions were also found in intermediate outcomes (blood pressure control in several conditions, and glycaemic control in diabetes)
No changes were observed for treatment indicators (influenza immunisation and antithrombotic treatment in coronary heart disease)

## Data Availability

English Quality and Outcomes Framework (QOF) data are publicly available, as are the Scottish QOF data before withdrawal of incentives. Scottish data on quality of care in the three years after QOF withdrawal (transitional quality arrangements data) can be obtained from Public Health Scotland. Code for performing the multiple group analysis is available at https://github.com/drzmorales/Abolishing-QOF-Incentives.

## Web extra

Extra material supplied by authors

Supplementary information: Figures S1 and S2 showing single group analysis of the 16 indicators for Scotland and England, respectively

## Notes

The lead author (DRM) affirms that the manuscript is an honest, accurate, and transparent account of the study being reported; that no important aspects of the study have been omitted; and that any discrepancies from the study as originally planned (and, if relevant, registered) have been explained.

Dissemination to participants and related patient and public communities: We will disseminate our findings to policy makers planning primary care fundings and services in the UK, to inform decision making on future Quality and Outcomes Framework in the UK. We will also share our findings with the public through press releases and social media engagement, and with the academic community at scientific conferences.

Provenance and peer review: Not commissioned; externally peer reviewed.
