# Supplementary material for: Estimated impact from the withdrawal of primary care financial incentives on selected indicators of quality of care in Scotland: controlled interrupted time series analysis
Source: BMJ. 2023 Mar 22;380:e072098. doi: 10.1136/bmj-2022-072098 (PMC10031759; doi:10.1136/bmj-2022-072098)
Supplement: Supplementary file 1 — Supplementary information: Figures S1 and S2 showing single group analysis of the 16 indicators for Scotland and England, respectively [file mord072098.ww.pdf]

Figure S1. Scotland single group analysis for the sixteen QOF indicators.

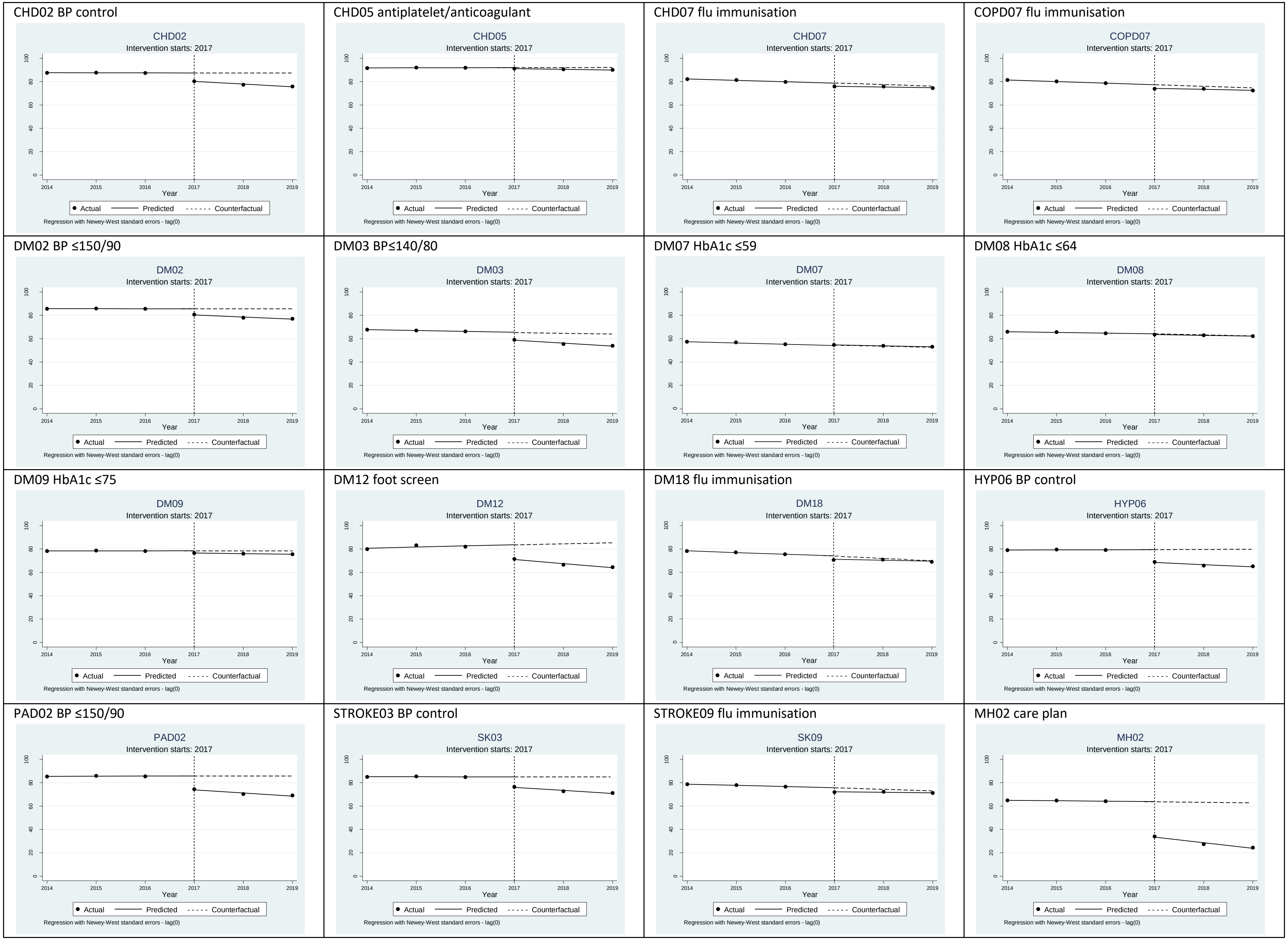

*See table 1 in the main text for definitions of indicators.

Figure S2. England single group analysis for the sixteen QOF indicators.

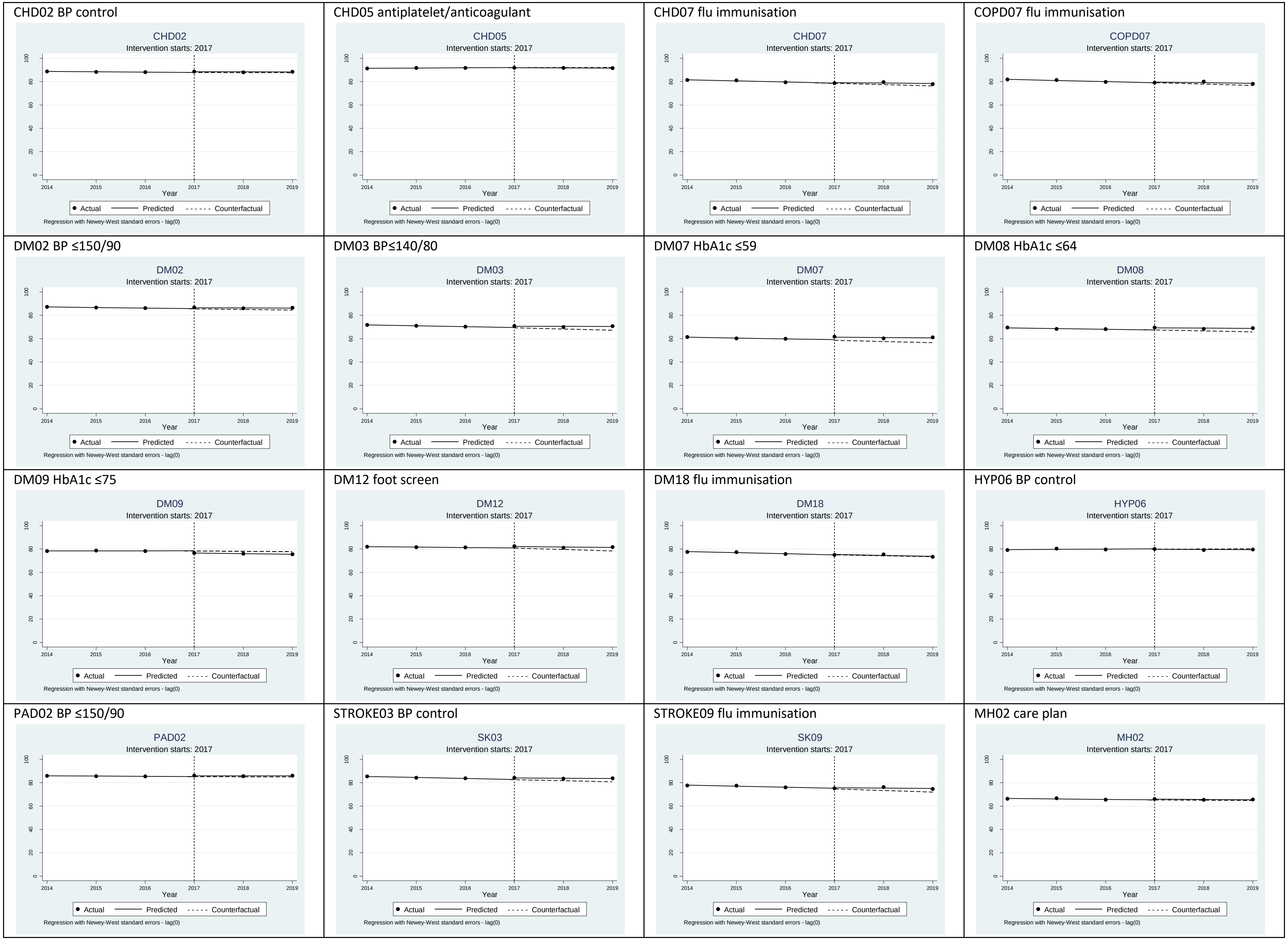

*See table 1 in the main text for definitions of indicators.
